# Endoscopic Ultrasound‐Guided Brachytherapy of Yttrium‐90 Implantation Into Pancreas: A Dose‐Escalation Pilot Study

**DOI:** 10.1002/mco2.70117

**Published:** 2025-02-24

**Authors:** Yuchong Zhao, Yilei Yang, Buchuan Zhang, Haochen Cui, Luyao Liu, Ronghua Wang, Yunfeng Han, Dongling Zhu, Wenliang Ma, Xinxing Zhang, Jinlin Wang, Si Xiong, Shuya Bai, Xiaohua Zhu, Bin Cheng

**Affiliations:** ^1^ Department of Gastroenterology and Hepatology Tongji Hospital, Tongji Medical College, Huazhong University of Science and Technology Wuhan China; ^2^ Department of Nuclear Medicine Tongji Hospital, Tongji Medical College, Huazhong University of Science and Technology Wuhan China; ^3^ Department of Surgery University of Pittsburgh School of Medicine Pittsburgh Pennsylvania USA; ^4^ Chengdu New Radiomedicine Technology Co., Ltd Chengdu China; ^5^ Department of Oncology Tongji Hospital, Tongji Medical College, Huazhong University of Science and Technology Wuhan China

**Keywords:** brachytherapy, endoscopic ultrasound, pancreatic cancer, porcine model, Yttrium‐90

## Abstract

Intratumoral brachytherapy enables higher dose treatment and reduces damage to adjacent tissues. We first validated the feasibility and safety of endoscopic ultrasound (EUS)‐guided Yttrium‐90 (^90^Y) microspheres implantation in a porcine model. Under EUS guidance, ^90^Y‐loaded microspheres were implanted into the pancreas of 10 miniature pigs. The first pig was implanted with 10 MBq particles. Subsequently, nine pigs were sequentially included in the low‐ (20 MBq), medium‐ (40 MBq), and high‐dose (60 MBq) groups. Positron emission tomography (PET)/CT imaging was used to check the occurrence of particle displacement postoperatively. After euthanasia, the pancreas and adjacent organs were excised for histological examination and residue radiation detection. The absorbed doses demonstrated safe in the porcine model were further in the xenograft model and *KRAS^LSL/+^Trp53^FL/FL^Ptfqa^Cre/+^* mouse model. EUS‐guided implantations of ^90^Y‐loaded microspheres were successful in all animals. Two pigs had mild serum amylase elevation in the high‐dose group and the abnormal index returned to baseline without interventions. The volume of necrotic lesions ranged from 255.76 to 745.57 mm^3^. In KPC mouse model, PET/CT imaging demonstrated a significant decrease in maximum standardized uptake value (SUVmax) after ^90^Y implantation. EUS‐guided ^90^Y‐loaded carbon microsphere implantation could serve as a safe and feasible technique at ultrahigh dose for pancreatic cancer brachytherapy.

## Graphical abstract

Under EUS guidance, the splenic lobe was located and ^90^Y‐loaded carbon microspheres were implanted into the pancreas of the Bama miniature pigs. Positron emission imaging/computer tomography (PET/CT) imaging was used to check the occurrence of particle displacement and radioactivity postoperatively. All pigs were euthanatized after 15 days postoperative. The pancreas and adjacent organs were excised for histological examination and residue radiation detection.

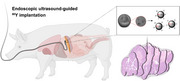

## 1 Introduction

The treatment options for pancreatic cancer remained limited during the past 20 years [1]. Recent evidence showed that the introduction of radiotherapy into the standard of care for borderline resectable pancreatic cancer (BRPC) or locally advanced pancreatic cancer (LAPC) could increase the R0 resection rate to 37%–82%, accompanied by improved overall survival (OS) and disease‐free survival (DFS), compared with upfront surgery or neoadjuvant mono‐agent chemotherapy alone [2, 3, 4, 5, 6, 7]. However, as a radiotherapy‐resistant type of tumor, only high‐dose or even ultrahigh‐dose radiotherapy could provide maximum benefit to patients with pancreatic cancer [4, 8]. Thus, it seems inevitable that external beam radiotherapy will result in radiation‐induced damage to surrounding hollow visera [9, 10]. Intratumoral brachytherapy enables higher dose radiation and controllable damage to neighboring healthy tissue [11].

EUS offers high‐accuracy, real‐time imaging and allows endoscopists to puncture the target within the pancreas. These characteristics of EUS make it irreplaceable for the diagnosis of pancreatic lesions [12]. Over the past 20 years, EUS has drawn increasing attention for its therapeutic potential. Compared with CT‐guided techniques, the EUS‐guided approach is less likely to result in bleeding, pain, and displacement [13]. EUS‐guided celiac plexus neurolysis, various modalities of ablation, and intratumoral radioactive particle implantation have been widely used for cancer pain control and palliative treatment for patients intolerant of surgeries. Iodine‐125 (^125^I) and phosphorus‐32 (^32^P) are the two types of currently used radionuclides in pancreatic cancer treatment [13, 14, 15]. ^125^I is mainly used for cancer pain palliation and ^32^P preliminarily shows its down‐staging potential in BRPC and LAPC [11, 16]. A pilot study of EUS‐guided ^32^P implantation for LAPC demonstrated that vascular involvement was no longer observed in six (50%) patients and 80% of patients underwent R0 resection with clear histological margins [11].

Yttrium‐90 (^90^Y) emits highly pure β‐particles and has moderate penetration within tissue. Compared to the same β‐particle emitting ^32^P, the half‐life period of ^90^Y is 64.5 h and that of ^32^P is 343.2 hours, which means that ^90^Y is able to release 87% of the energy in 7 days while ^32^P requires 42 days [17, 18]. Besides, the maximum and mean energy of ^90^Y (2.27 MeV, 0.94 MeV) are both higher than those of ^32^P (1.71 MeV, 0.59 MeV) [17, 19]. These merits enable higher energy to kill tumor cells in a short period of time, and the radioactive waste produced during the medical procedure or in patients’ feces can be released from control in a shorter duration. Another critical advantage of ^90^Y is that it can be imaged through internal pair production with PET imaging. Currently, ^90^Y is considered as a promising option for liver cancer treatment via vascular intervention. Through intratumoral implantation, it is expected to generate greater resistance during injection compared to vascular intervention [19, 20]. To make the particles easier to pass through the 22‐gauge needle during EUS and avoid needle obstruction, we substituted the traditional glass, resin microspheres, or silicon particles with novel biocompatible, but non‐biodegradable carbon microspheres. This pilot study evaluated the feasibility and safety of EUS‐guided ^90^Y‐loaded carbon microsphere implantation in a porcine model. Following this, the study further evaluated the effects of intratumoral ^90^Y on the growth of solid tumor growth in an in vivo xenograft model and a KPC mouse model.

## 2 Results

### 2.1 EUS‐Guided ^90^Y Implantation Into Pancreas

**FIGURE 1:**
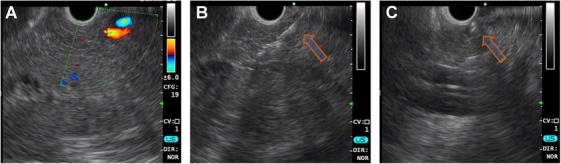
EUS‐guided 90Y microspheres implantation. (A) The splenic lobe was located under EUS guidance. (B) The needle was punctured into the pancreas and ^90^Y microspheres were injected. (C) After implantation, a hyperechoic region was observed in the implanted area.

**FIGURE 2:**
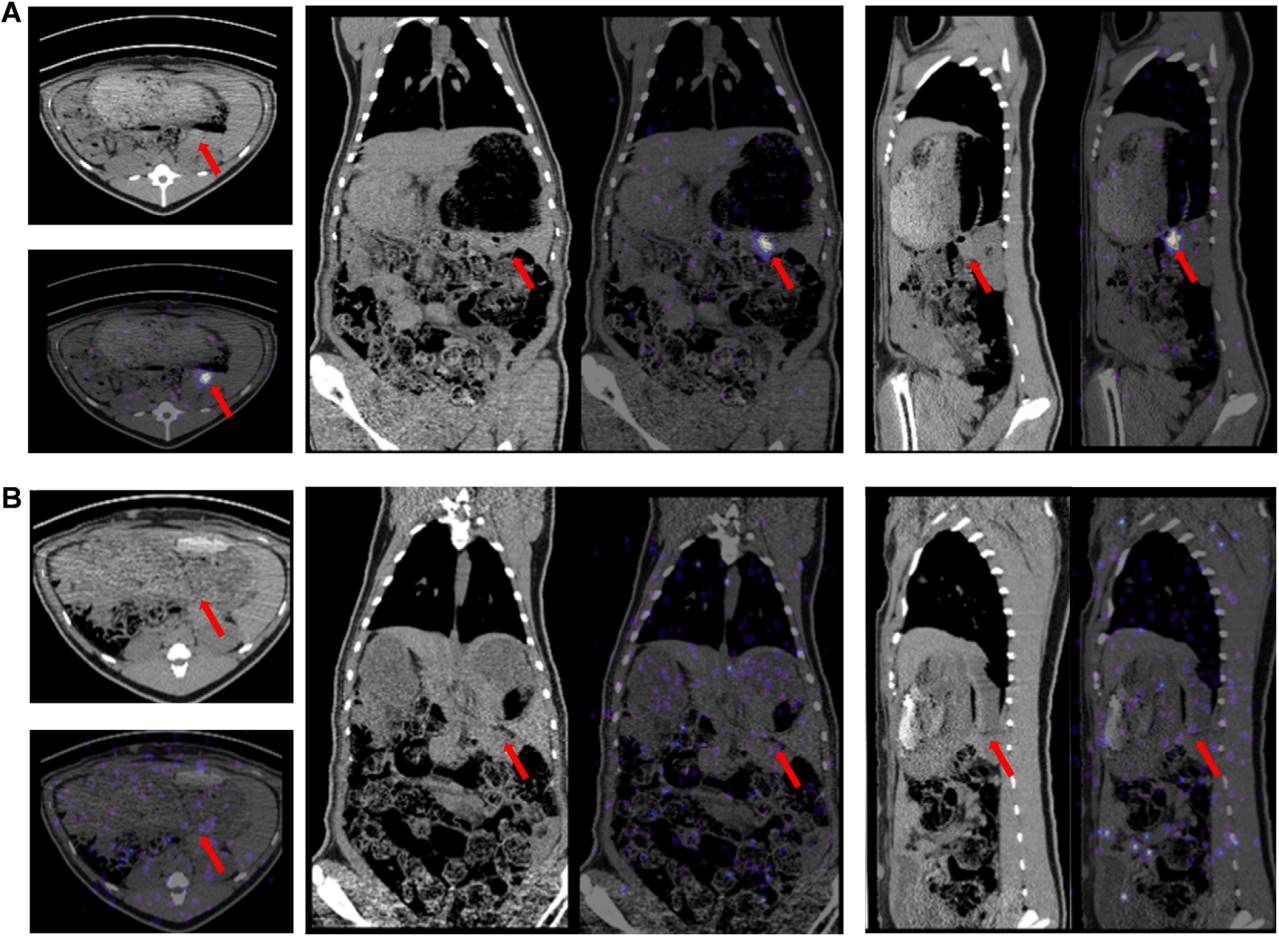
Postoperative PET/CT imaging of pig No.9 (60 MBq). (A) The injection site in pancreas showed high uptake at 4 h postoperatively (pancreas: red arrow) (left: transverse plane; middle: coronal plane; right: sagittal plane). (B) No uptake signal was observed in the pancreas at 14 days postoperatively (left: transverse plane; middle: coronal plane; right: sagittal plane) (pancreas: red arrow).

Under real‐time EUS‐guided visualization (Figure 1 and Video S1), ^90^Y‐loaded carbon microspheres were injected into the splenic lobe of the pancreas (corresponding to the tail and body of the human pancreas). All operations were completed and no needle obstruction‐induced implantation failure occurred. During the injection, a hyperechoic irregular area with mild dispersion appeared around the distal tip of the needle. Obvious dispersion occurred in one animal from the high‐dose group. PET/CT imaging confirmed that the radioactive signal was localized within the pancreas body‐tail in all pigs on the day of implantation, as well as at 3 days (Figure 2A and Figure S1). In addition, only one pig (10%) showed a detectable radioactivity signal in the lower abdominal on postoperative Day 3. No visible signals in non‐injected areas were detected in the remaining pigs, indicating no obvious migration of the particles. No uptake signal was observed in the pancreas 14 days after surgery on PET/CT imaging (Figure 2B). After euthanasia, the para‐lesion pancreas, distal pancreas, spleen, left kidney, and liver were excised for radioactivity examination. The radioactivity of the pancreas parenchymal adjacent to the necrosis (130.1 ± 70.49 Bq) was not significantly higher than that of any other sites and there were no intergroup differences (Figure S2). The radioactivity detected in urine, stools, and blood samples was not significantly higher than background radioactivity (Table S1).

### 2.2 Safety and Tolerability

**FIGURE 3:**
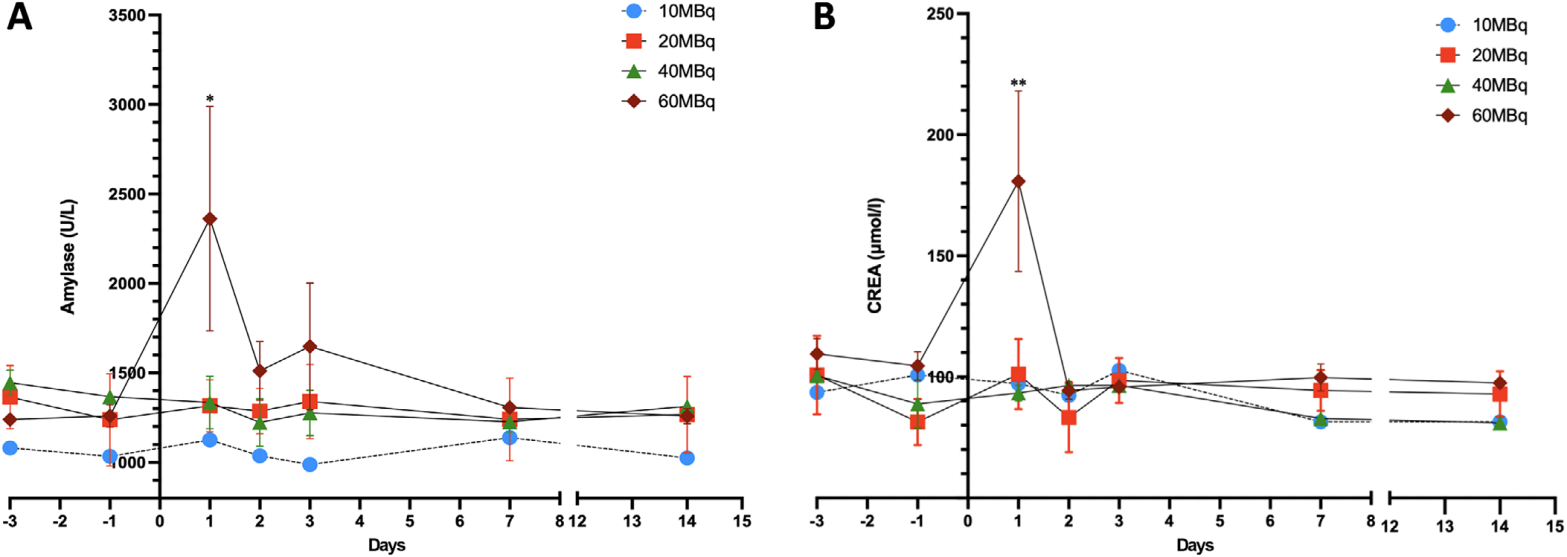
Abnormal serum index. (A) Serum amylase levels in the high‐dose group elevated significantly postoperatively and returned to baseline on the seventh day. (B) CREA levels elevated significantly on the first day postoperatively and returned to baseline the next day. *p < 0.05, **p < 0.01.

All pigs tolerated the procedure well and survived to the end of the follow‐up period. One pig in the high‐dose group showed elevated amylase levels (3616.14 U/L) 24 h after the operation. However, the amylase level was back to normal on the seventh day without specific intervention. The serum amylase level in the high‐dose group was significantly higher than the preoperative amylase baseline (2362.364 ± 627.089 vs. 1239.774 ± 36.817 U/L, *p* = 0.007) (Figure 3A). A moderate elevation of aspartate aminotransferase (AST) to 98.30 U/L (3.8 times higher than the preoperative baseline) on Day 1 postoperative was observed in the same pig. In addition, the creatine (CREA) levels of two pigs from the high‐dose group rose significantly to 227.14 and 208.06 µmol/L, but returned to the baseline by the next day postoperatively (180.784 ± 37.246 vs. 109.500 ± 6.318 µmol/L, *p* = 0.001) (Figure 3B). None of the pigs in the medium‐ or low‐dose groups exhibited clinical symptoms, including fever, inappetence, irritation, or laboratory‐proven abnormalities. The postoperative red blood cell count, white blood cell count, platelet count, alanine aminotransferase (ALT), total bilirubin (TBIL), direct bilirubin (DBIL), γ‐glutamyl transpeptidase (γ‐GT), glucose (GLU), and urea nitrogen (UREA) were within the normal range (Figure S3). Anatomic observation and CT imaging showed no signs of pseudocyst formation and free fluid around the pancreas or in the abdominal cavity. These results preliminarily demonstrate the safety and tolerability of EUS‐guided ^90^Y implantation into the pancreas.

### 2.3 Pathological Observation

**FIGURE 4:**
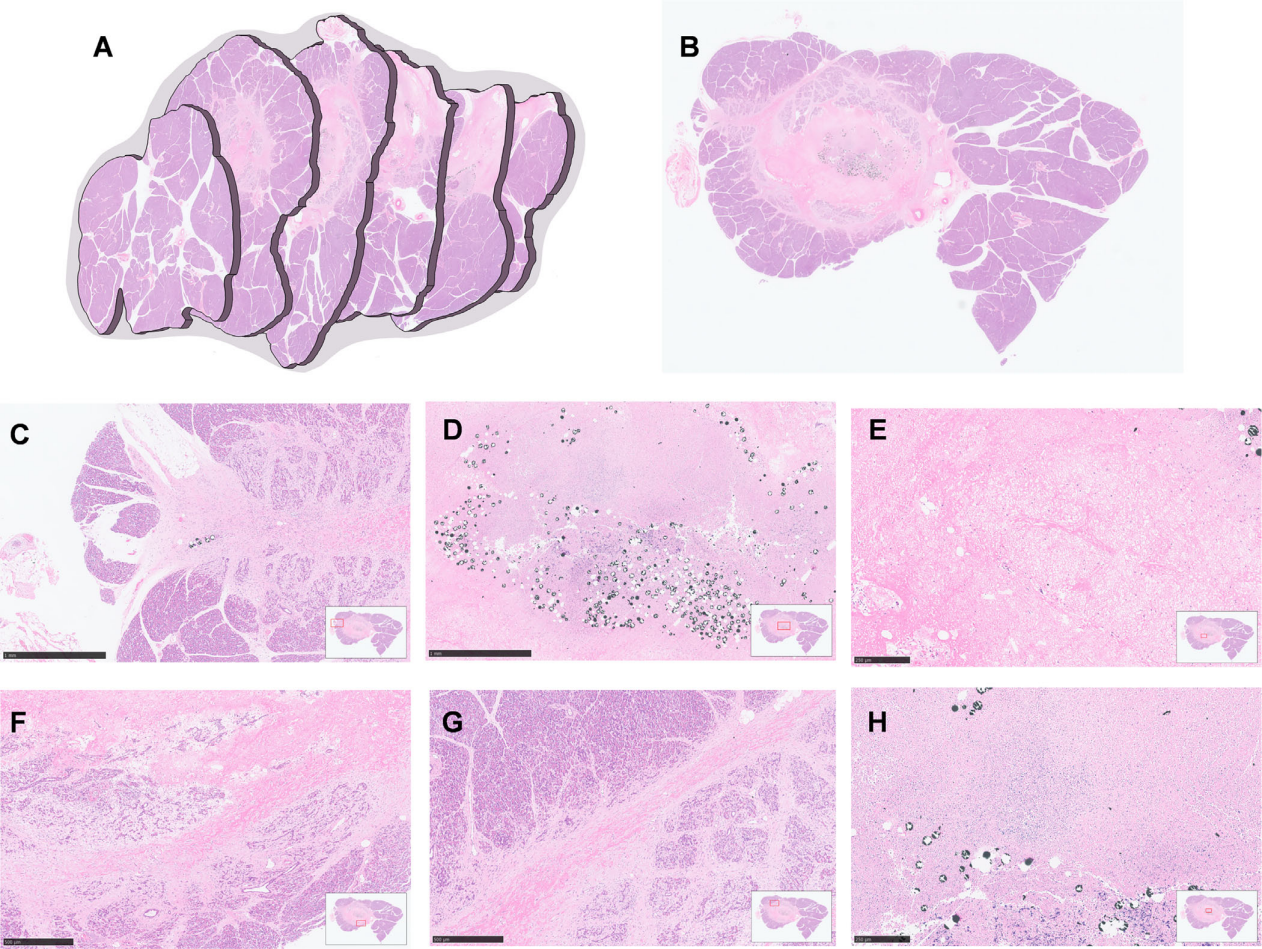
Specimen from a histopathological examination. (A) Three‐dimensional radiation‐induced necrosis reconstruction. (B) Carbon microspheres (diameter ranged from 25 to 50 µm) observed in the necrosis core. (C) A few carbon microspheres were observed in the needle pass. (D) Suppurative inflammation in the necrosis core. (E) Coagulation necrosis in the peripherals of the lesion. (F–G) Fibrosis and collagen exudation in the peripherals of the lesion. (H) The outer layer of the lesion and an exhibiting of coagulation necrosis and inflammatory cells distributed within.

The excised pancreas was cut into 0.3–0.5 cm slices from tail to head, and the area localized within 2 cm of diameter within the macroscopic margin of the necrotic lesion was further sliced at consecutive levels of 500 µm (Figure S4). The cores of the treated areas were visualized as an elliptical necrotic lesion with a long diameter of 9.29 ± 0.75 mm (95% confidence interval [CI] 8.35–10.22 mm) and a short diameter of 6.26 ± 0.95 mm (95% CI, 5.08–7.44 mm) upon gross observation. The periphery of the necrosis had an irregular outline. Grayish‐black and white necrotic areas were surrounded by healthy pink pancreatic tissue. A hemorrhage or ecchymosis borderline of 0.2–0.4 mm around the lesion could be observed. HE‐stained specimens showed a clear boundary between the necrotic area and the healthy pancreas (Figure 4A). Carbon black microspheres can be observed in the core of necrosis and along the needle tract within the pancreas (Figure 4B,C). Histopathological examination revealed a core area containing microspheres encapsulated in coagulated necrosis. In the medium‐ and high‐dose groups, the necrosis was surrounded by a rim of exudative collagen and fibrosis. However, the fibrosis rim was not evident in the low‐dose group (Figure 4D–H). Inflammatory cells were distributed in the peripheral parts of the necrotic and fibrotic lesions. The mean cross‐section area of the necrosis was 51.99 ± 10.19 mm^2^ (95% CI, 44.70–59.28 mm^2^) and the mean volume of the core necrosis lesion was 505.85 ± 216.45 mm^3^ (95% CI, 237.09–774.61 mm^3^) with no significant differences among the groups (Figure S5). No histological abnormalities were identified in the adjacent stomach, left kidney, liver, or spleen. These findings elucidated the radiation‐induced lesion range and further indicate the safety of ^90^Y implantation.

### 2.4 Intratumoral ^90^Y Implantation in Pancreatic Carcinoma Xenograft and KPC Mouse Model

**FIGURE 5:**
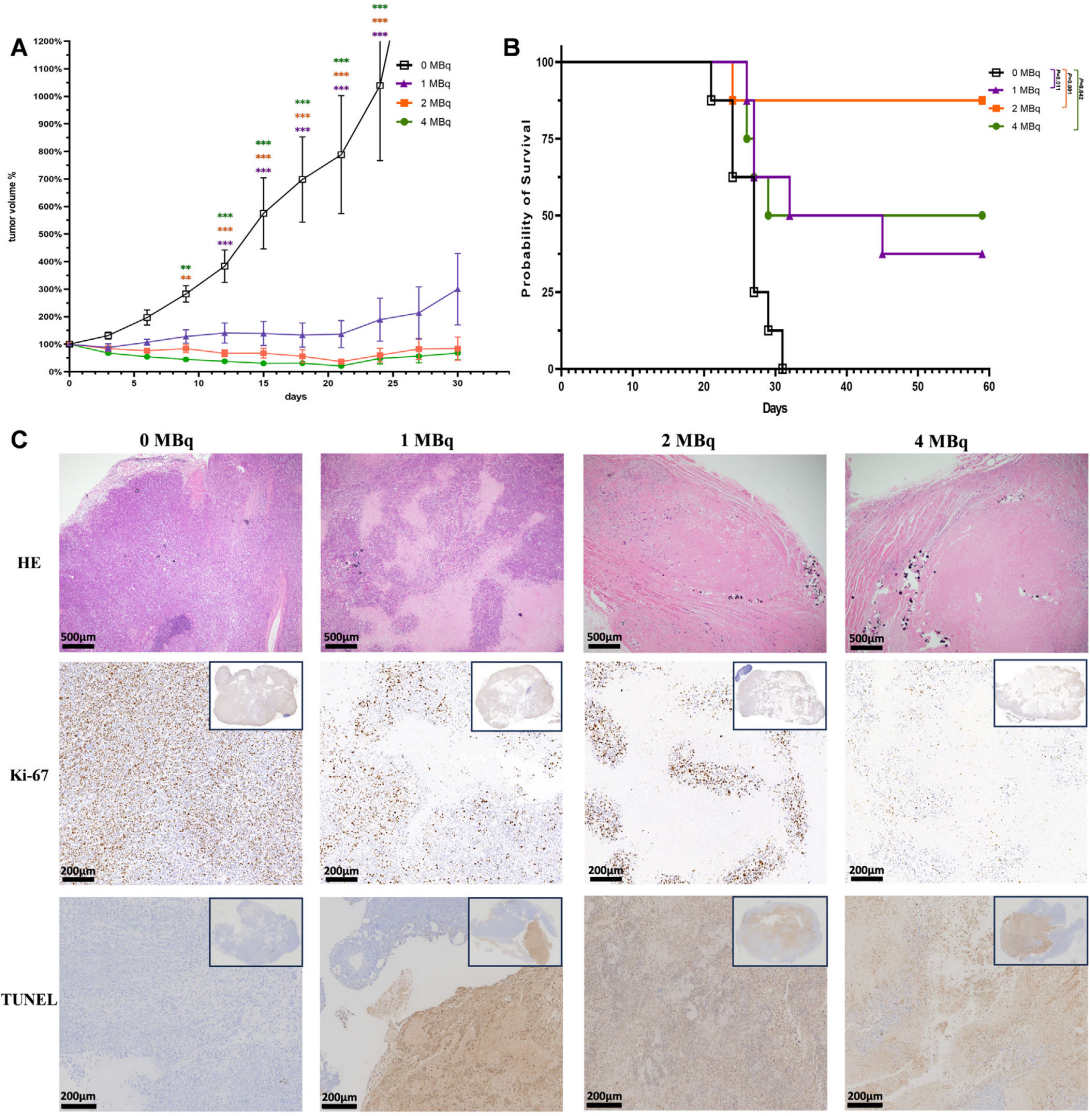
Assessment of the effect of ^90^Y in PANC‐1 xenograft model. (A) ^90^Y implantation suppressed the growth of pancreatic cancer cell line PANC‐1 xenografts in nude mice. (B) ^90^Y implantation significantly prolonged the OS of nude mice in three treatment groups compared with the control. (C) Complete responses were observed in 2 MBq and 4 MBq treatment groups. High dose of radiation was correlated with a decreased ki‐67 index and increased apoptosis. **p < 0.01, ***p < 0.001.

The safety and tolerability of 100, 200, and 400 Gy regimens were validated in the porcine model. These three doses were subsequently evaluated in xenograft and KPC mouse models. Through a radiation clonogenic assay, AsPC‐1 was identified as the most radioresistant cell line and PANC‐1 as a radiosensitive candidate (Table S2 and Figure S6A,B). When the diameter of subcutaneous xenografts reached 10 mm, 1, 2, or 4MBq of ^90^Y‐loaded microspheres or empty microspheres were implanted into the cores of the xenografts. ^90^Y implantation notably suppressed tumor growth and prolonged the OS in all three dose groups (Figure 5A,B and Figure S6C,D). Mice in the radiosensitive PANC‐1 group benefited more from the ^90^Y radiation therapy, especially in the 200 and 400 Gy groups. At the 2‐month endpoint, 87.5% of mice in the 2 MBq PANC‐1 group were still alive. However, there were no statistical differences in growth rate and OS among the three dose groups. HE staining showed coagulation necrosis in the core of the tumor (Figure 5C and Figure S6E). Notably, in the PANC‐1 model, two (25.0%) in the 2 MBq group and three (37.5%) in the 4 MBq group achieved complete remission (complete response is defined as the histologic examination of implantation sites shows no viable tumor tissues). No complete response was observed in the AsPC‐1 models. Xenografts were analyzed for cell proliferation and apoptosis through immunostaining for ki‐67 and TUNEL [21, 22]. IHC staining demonstrated that ^90^Y significantly decreased the expression of ki‐67 and mice that received ^90^Y implantation had significantly more apoptosis in the xenograft tumors than controls (Figure 5C and Figure S6E). The escalation of the radiation dose was positively correlated with the suppression of cell proliferation.

**FIGURE 6:**
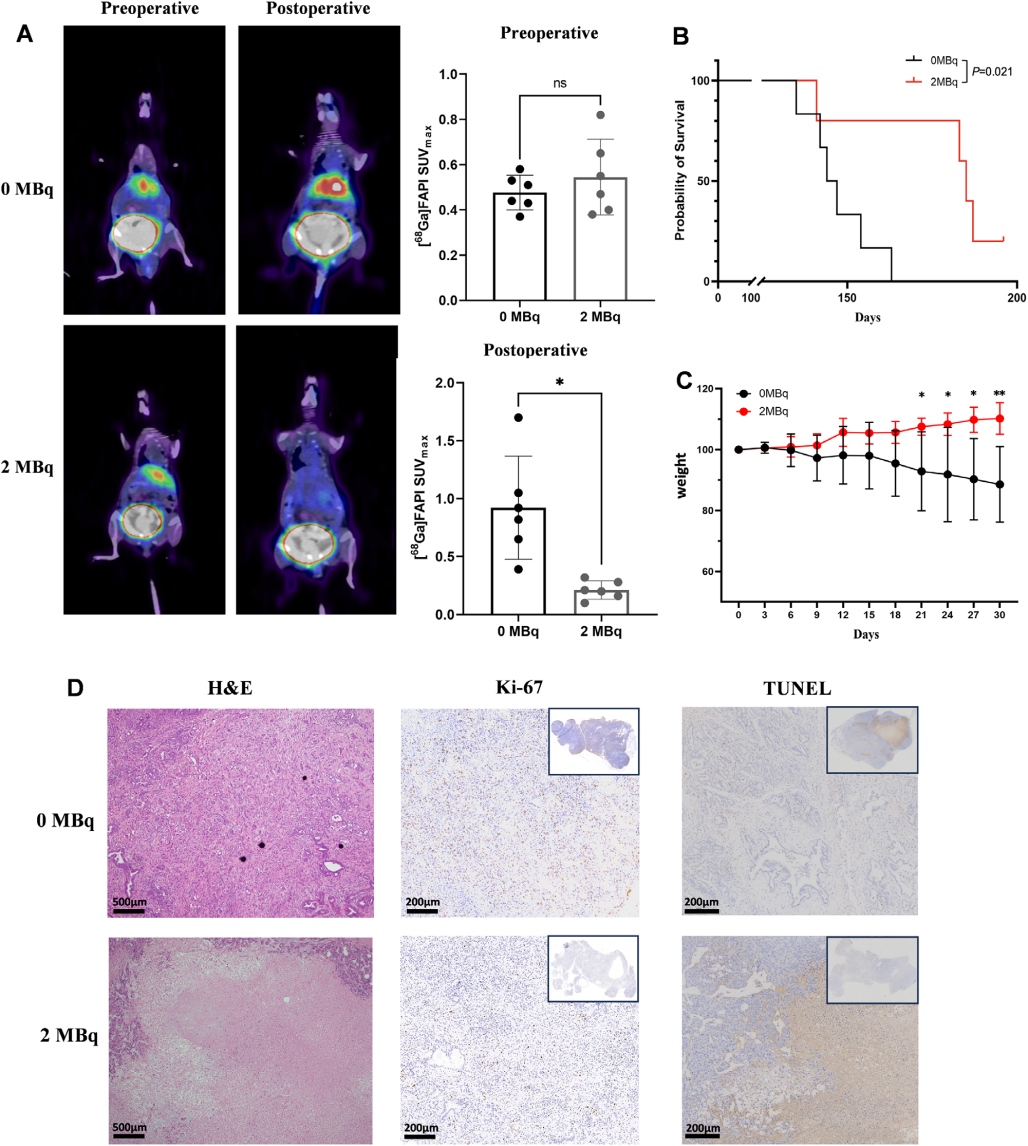
Intratumoral ^90^Y implantation in KPC mouse model. (A) After ^90^Y implantation, the SUVmax of KPC mice in the treatment group was significantly lower than the control group. (B) KPC mice accepted ^90^Y implantation had better overall survival compared with the control. (C) The weight gain of mice in the treatment group was significantly higher than those in the control group. (D) Radiation‐induced necrosis could be observed in the tumor of KPC mice accepted ^90^Y implantation. IHC for ki‐67 and TUNEL showed a decrease in proliferation and an increase in apoptosis. *p < 0.05, **p < 0.01.

Twelve sex‐matched 18‐20‐week‐old KPC mice were randomly divided into two groups. Preoperative PET/CT imaging demonstrated the presence and locations of the tumor. There were no differences in tumor sizes and the baseline maximum standardized uptake value between the two groups of KPC mice (SUVmax). According to the outcomes in the xenograft nude mouse trial, a 200 Gy regimen was adopted in the KPC mouse model. Note that 2 MBq of ^90^Y‐loaded or empty microspheres were implanted into the lesions within the pancreas. Compared to the control group, PET/CT imaging on the 14th day demonstrated ^90^Y implantation significantly decreased SUVmax of the tumor (0.212 ± 0.090 vs. 0.922 ± 0.497, *p* = 0.014) (Figure 6A). Subsequent survival analysis showed that the intratumoral radiation prolonged the OS and the body weight of the control group significantly decreased compared with the ^90^Y group (Figure 6B,C). IHC staining showed radiation‐induced necrosis in the tumor and significantly decreased ki‐67 expression and more apoptosis of the ^90^Y group KPC mice (Figure 6D), which further indicated that intratumoral ^90^Y implantation as a promising approach for pancreatic cancer treatment.

## 3 Discussion

To increase the chance of R0 resection and improve locoregional control, radiation therapy has been integrated into the treatment of patients with BRPC and LAPC without systemic metastasis [23, 24]. However, the optimal radiotherapy approach is still under debate due to mixed results. Randomized controlled trials including the 2022 PREOPANC trial and the 2023 ESPANC‐5 trial demonstrated a higher R0 resection rate and better OS with chemoradiation regimens compared to upfront surgery in BRPC patients [2, 6, 25]. In contrast, the ALLIANCE A021501 trial found no extra benefits of stereotactic body radiotherapy for the multiagent chemotherapy regimen [9]. Besides, the addition of radiotherapy led to increased grade 3 or higher adverse events. Although most of the findings favor the paradigm of preoperative chemoradiation being associated with better locoregional disease control and prolonged survival for BRPC and LAPC patients, the role and type of radiotherapy for BRPC and LAPC remain controversial. In summary, the radiation dose has been identified as an independent factor affecting OS and local disease control [4]. Ongoing trials using high‐dose radiotherapy techniques, for example, SBRT, and stereotactic ablative body radiotherapy (SABR) are all attempting to escalate the radiation doses to achieve better local targeting [1, 8]. Although pancreatic cancer is insensitive to radiation, recent studies have demonstrated that radiotherapy could activate immune effectors against various gastrointestinal tumors, including pancreatic cancer [26, 27, 28]. Therefore, we carried out this pilot study to evaluate the feasibility and safety of brachytherapy enabling both potentially higher radiation dose and reduced toxicity.

^125^I has been used in the palliative treatment of pancreatic cancer‐induced pain for a long time. A new radioactive agent, ^32^P microparticles (Oncosil, Sydney, Australia), administrated through EUS‐guided implantation, has been approved for unresectable pancreatic cancer treatment. The PanCO study recruited 42 patients and 10 patients (23.8%) underwent resection after the combination of chemotherapy and ^32^P implantation, with eight (80%) achieving R0 resection [16]. Another pilot study also demonstrated the value of these radioactive particles [11]. After two cycles of chemotherapy, intratumoral ^32^P implantation achieved tumor downstaging in six LAPC patients (50%) and successful resection in five patients (42%), including four R0 resections (80%). Apart from better locoregional response, pain control, tumor volume reduction, metabolic changes, and CA19‐9 decline were also observed following particle implantation. These encouraging results suggest that intratumoral implantation of radioactive particles could be a promising therapeutic approach for unresectable pancreatic cancer patients. In this pilot study, we demonstrated the localization of ^90^Y to the injected site with only minimal systemic distribution in a porcine model. The absorbed dose validated as safe and effective in killing tumor xenografts was further evaluated in KPC mouse model, where the median OS of KPC mice was significantly prolonged after ^90^Y implantation.

Transarterial ^90^Y radioembolization significantly prolongs the progression‐free survival compared with chemoembolization in patients with advanced hepatocellular carcinoma [19]. Several guidelines have recommended transarterial ^90^Y radioembolization for patients with unresectable hepatocellular carcinoma and colorectal carcinoma metastasis to the liver. ^90^Y is a highly pure β‐particle emitter. Thus, the radiation risk to the surrounding environments from ^90^Y implantation is significantly lower than that of the γ‐particle emitting ^125^I. Compared to the similar β‐particle emitting ^32^P, ^90^Y has a slightly longer penetration within tissue (Rmax: 11 mm vs. 8 mm) and greater maximum energy (2.27 MeV vs. 1.71 MeV) [18, 29]. The major advantage of ^90^Y is that its half‐life period is 64.05 h compared to the 14.27 days for ^32^P, which means the self‐isolation period can be lifted much sooner after ^90^Y implantation. Besides, ^90^Y decays into stable and non‐toxic zirconium, whereas ^32^P decays into more active sulfur. Considering these factors, we believe that ^90^Y has the potential to act as a preferred therapeutic option for pancreatic cancer once the issue of the administration route is settled. Traditional vehicles for ^90^Y include resin or glass spheres and the vehicle used in Oncosil is composed of multiform silicon particles. To facilitate the ^90^Y injection, we selected a lower density medium, carbon microspheres as the vehicle. In our study, all procedures succeeded, and no needle obstruction occurred. In one animal, we observed obvious dispersion during the injection in the high‐dose group, possibly due to the elevated concentration of the agents. However, the dispersion was localized in the other animals. These results indicate the feasibility of ^90^Y implantation through EUS‐guided implantation.

In the PanCO study, a 100 Gy absorbed dose of ^32^P, calculated from the tumor volume was delivered [16]. We selected 100 Gy as the starting point of our dose‐escalation study to evaluate the safety of the operation. Two (66%) animals in the high‐dose group (60 MBq–600 Gy) had elevated serum amylase and CREA levels. Notably, we implanted the ^90^Y‐loaded microspheres into normal pancreas, and the pancreatic duct was directly involved in the radiation. Under real clinical circumstances, the radioactive particles would be implanted in a tumor normally greater than 2 cm. Thus, we assume that adjacent surrounding tissue or viscera are less likely to be involved. No clinical symptoms or laboratory‐proven abnormalities were observed in the medium and low‐dose groups. These two dosages are still higher than most of the currently utilized clinical standards. The monitoring of leakage dose revealed that the radioactivity in urine, stool, and blood samples was not higher than that in the environment. The radioactivity of excised adjacent organs was also within an acceptable range, indicating that the particles rarely leak into the circulation. Histopathological results demonstrated that the carbon microspheres localized at the puncture site and did not disperse. The range of the radiation‐induced necrosis diameter was 10–13 mm, and the necrosis was surrounded by a rim of fibrosis. The clear borderline indicated limited radiation toxicity to adjacent healthy tissues. Manifestations of pancreatitis occurred when the absorbed dose escalated to 600 Gy. However, the necrosis volumes were not significantly different among the groups. The possible reason for the adverse events may be due to an increase in leakage through the puncture route. We need to adapt to multi‐spot implantation within the tumor to avoid possible leakage by decreasing the dosage per injection. Overall, the findings demonstrated that the implantation of a commonly used dose was safe.

The limitations of this dose‐escalation study are its relatively small sample size, lack of prolonged follow‐up duration, and that the operation was performed through a single route in the normal pancreas, not in the tumor. However, this pilot study provides a novel therapeutic approach for pancreatic cancer patients through the use of ^90^Y encapsulated in lower density carbon microspheres. Second, radiotherapy was used as a single regimen in the KPC mice treatment, without combination with chemotherapy or immunotherapy, which may not be the optimal modality for pancreatic cancer treatment.

## 4 Conclusions

The ^90^Y microsphere implantation under EUS guidance was found to be safe and feasible. A higher dose of radiation could enable a greater chance of locoregional control and better survival outcomes. We hope this pilot study can offer a framework for further phase I/II studies.

## 5 Materials and Methods

### 5.1 Porcine Model

Ten healthy Bama minipigs weighing 30–45 kg were individually housed according to guideline standards and were observed for 1 week before EUS‐guided implantation. The first pig was used for the procedure training, estimating the residual dose in the endoscope, and as the start of dose escalation. A total of 10 MBq radioactive microspheres were implanted in the pancreas. When the procedure succeeded and no severe complications were noted, the other nine pigs were sequentially divided into the low‐dose group (20 MBq), medium‐dose group (40 MBq), and high‐dose group (60 MBq).

### 5.2 ^90^Y‐Loaded Carbon Microspheres

The radioactive yttrium chloride was created through thermal neutron capture within a nuclear reactor, which transmutes the natural yttrium (^89^Y) in multiaperture carbon microspheres (Figure S7) to the β‐emitting radionuclide ^90^Y. The theoretical maximum penetration distance within tissue is 11 mm. ^90^Y‐loaded microspheres were produced 3 days before the procedure and the total radioactivity was 1 GBq/mL at the time of calibration. The microspheres had an average particle size of 32 µm, with a size range of 20–50 µm. The microspheres were then filled and sterilized at NRT Technology (Chengdu, China). When EUS localized the injection point, ^90^Y particles were immediately measured by a radioisotope dosage calibrator (CRC‐15, Capintec, NJ, USA), and different doses of ^90^Y were dissolved in a sterile sodium carboxymethyl cellulose solution. The following formula was used to calculate the total absorbed dose after decay:

$$D\left[\mathrm{Gy}\right]=49.9\frac{A_{0}\left[\mathrm{GBq}\right]}{m\left[\mathrm{kg}\right]}$$

Assuming the tumor was a sphere with a 2 cm diameter and a density of 1.05 g/cm^3^, the starting start dose for the dose‐escalation study was 100 Gy, considering the residua dose and multiplying the theoretical radioactivity by a coefficient of 1.2, the required radioactivity was calculated to be 10.57 MBq. Thus, the absorbed doses were 200 Gy in the low‐dose group, 400 Gy in the medium‐dose group, and 600 Gy in the high‐dose group, respectively. The injection volume of the ^90^Y solution was 8% of the assumed tumor volume and was estimated to be 300–350 µL. The actual radioactivity of ^90^Y microspheres implanted in each pig is listed in Table S3.

### 5.3 Pre‐Procedure Preparations and Endoscopic Procedures

All pigs underwent fasting for 12 h before the procedure. Zoletil*^®^* 50 (5mg/kg) was administered subcutaneously for induced anesthesia. After intubation, propofol was administered intravenously to maintain general anesthesia, and oxygen was given via a ventilator to maintain respiration. Cardiac and respiratory rates were monitored throughout the procedure. An Olympus EU‐ME1 US system (Olympus, Tokyo, Japan) and GF‐UCT 260 linear‐array echoendoscope (Olympus, Tokyo, Japan) were used for the procedure. All operators wore lead clothing and radioactive contamination was monitored by a Geiger counter. The pyloric muscle of pigs was too thick for the endoscope to pass through and the splenic lobe was the widest part of a pig's pancreas. Thus, the transgastric EUS procedures were directed toward the splenic lobe of the pancreas, corresponding to the body and tail of the pancreas in humans. Under Doppler visualization, puncture and implantation were performed with a 22‐gauge needle (Cook, Winston‐Salem, USA). When the splenic lobe of the pancreas was localized, the needle was punctured into the target. The needle stylet was removed and attached to a T‐shaped tube. The other two ends of the tube were connected to a 1‐mL syringe loaded with ^90^Y microspheres and a 1‐mL saline pre‐flushed syringe. Firstly, the ^90^Y syringe was slowly pushed to the end, following by pushing the saline pre‐flushed syringe to the end. Finally, the ^90^Y syringe was pushed to the end again. The valve was rotated to the corresponding position after each injection. To wash out as many residual radioactive particles from the syringe and endoscope into the target as possible. The needles were retrieved and washed repeatedly, and the wasted water was examined for residual radioactivity.

### 5.4 Postoperative Procedures and Implantation‐Induced Necrosis Evaluation

PET/CT imaging data were collected on a PET/CT scanner (uBio‐Explorer, United Imaging, China) at multiple time points after surgery. PET/CT imaging was performed 4 h, 72 h, and 14 days after the procedure to check for the localization and occurrence of dispersion of ^90^Y. Data were reconstructed to obtain higher‐resolution images using an ordered subsets expectation maximization (OSEM) algorithm (8 OSEM iterations) with scatter, attenuation, and decay corrections applied.

Serum levels of amylase, ALT, AST, TBIL, DBIL, UREA, CREA, GLU, and γ‐GT were assayed preoperatively on day 3 and 1 and postoperatively at day 1, 2, 3, 7, and 14. To determine the potential leakage into the circulation and gastrointestinal tracts, the urine and stool samples were collected on preoperative day 1 and postoperative day 1, 2, 3, 7, and 14. The samples were examined using a liquid scintillation counter (Tri‐carb 4910TR, PerkinElmer, Waltham, USA). After 14 days of observation, the animals were euthanized by intravenous injection of pentobarbital (100 mg/kg). Animals were autopsied to exclude the occurrence of pancreatic pseudocyst, abdominal hemorrhage, fistula, or ascites. The pancreas was excised to observe the response to brachytherapy. The lesion sites were cut into slices to evaluate the pathological findings of brachytherapy by one pathologist (D.Y.). For gross pathological observation, visible necrosis was measured with calipers in fresh tissues before being preserved in 4% paraformaldehyde and H&E stained. The necrosis area of each slide was calculated using the NDP.view2 software (Hamamatsu, Japan), and the volume of each lesion was calculated by using the sum of the necrosis area on each slide multiplied by the thickness of the slide. The para‐lesion pancreas, left kidney, liver, and spleen were also excised and cut into a 1 cm^3^ cube, dissolved into tissue homogenate using a high‐speed, low‐temperature tissue grinder (Servicebio, China), and examined for the residual radioactivity by a liquid scintillation counter (Tri‐carb 4910TR, PerkinElmer, Waltham, USA).

### 5.5 Cell Culture

The human pancreatic cancer cell lines AsPC‐1 and BxPC3 were obtained from the American Type Culture Collection (ATCC, Manassas, USA). PANC‐1, Capan2, and SW1990 were purchased from the Cell Resource Center, Institute of Biochemistry and Cell Biology at the Chinese Academy of Science (Shanghai, China). All cell lines were cultured in Dulbecco's modified Eagle's medium (DMEM; GIBCO, NY, USA) supplemented with 10% fetal bovine serum (FBS; GIBCO) at 37°C and 5% CO_2_. Cells were confirmed to be free of mycoplasma using the Bimake mycoplasma detection kit.

### 5.6 Radiation Clonogenic Survival Assay

Cells were seeded on six‐well plates at a density of 3 × 10^2^, 5 × 10^2^, 1 × 10^3^, and 2 × 10^3^ cells per well and exposed to irradiation at doses of 0, 1, 2, 4, and 6 Gy, respectively. After incubation for 14 days at 37°C, the plated cells were washed with PBS, fixed with 4% paraformaldehyde for 30 min, and stained with crystal violet for 20 min. Colonies (≥ 40 cells/colony) were counted under a dissecting microscope. The surviving fraction (SF) curve was fitted using via a multi‐target single‐hit model with the following formula: SF = 1 − (1 − *e* ^−D/ D0^)^N^. The experiment was performed three times with replicates in each experiment.

### 5.7 Immunohistochemistry

The xenograft tumor from nude mice and pancreatic tissues from KPC mice were harvested as described previously for all analyses [30]. For ki‐67 analysis, slides were dewaxed and rehydrated, and treated with sodium citrate used for antigen retrieval. Slides were rinsed and blocked with 2.5% goat serum for 1 h then incubated with a ki‐67 antibody (CST; Rabbit; 9129; 1:400) at 4°C overnight in a humidified chamber. Following incubation with the primary antibody, slides were incubated with peroxidase‐conjugated secondary antibody and developed with DAB Substrate Kit (all from Vector Laboratories). Slides were counterstained with hematoxylin, dehydrated, and mounted with Permount. TUNEL assay was performed with In Situ Cell Death Detection Kit (Roche; 11684817910) as the manufacturer's protocol.

### 5.8 Mice and Tumor Models

Sex‐matched male and female nude, 8‐week‐old nude mice (Balb/c) were housed under institutional pathogen‐free conditions on a 12‐h reverse light–dark cycle. To establish xenograft tumor model, 1 × 10^7^ of AsPC‐1 or PANC‐1 cells was subcutaneously injected into the right flank of Balb/c mice. Upon reaching a tumor diameter of 1 cm, the nude mice were randomly assigned to groups receiving doses confirmed to be safe in the porcine model (1 MBq: 125 Gy; 2 MBq: 250 Gy; 4 MBq: 500 Gy) and a control group. ^90^Y‐loaded microspheres or empty microspheres were injected in the center of the tumors at a depth of about 4 – 5 mm. Tumor volume (*V*) was evaluated every 3 days, up to 2 months, by measuring the short axis (*W*) and the long axis (*L*) of the xenograft and calculated using the following formula: *V = 1/2 × L × W^2^*. *KRAS^LSL/+^* and *Trp53^FL/FL^*; *Ptfqa^Cre/+^* mice were backcrossed to a pure C57BL/6 background over 10 generations and then bred to each other to produce the *KRAS^LSL/+^*, *Trp53^FL/FL^*, and *Ptfqa^Cre/+^* animals. Genotyping was performed as previously described [31]. At 16 weeks of age, KPC mice were subjected to weekly screening for tumors by brief exposure to inhaled anesthesia, followed by abdominal palpation. Mice with any suspected lesion on palpation were anesthetized with 2% isoflurane and subjected to ^68^Ga‐DOTA‐FAPI micro‐PET/CT imaging. ^8^Ga‐DOTA‐FAPI uptake was quantified by drawing region of interest (ROI) using IRIS PET/CT software (United Imaging, China) and plotting maximum standardized uptake values (SUVmax). According to the imaging indicating, age‐ and sex‐matched tumor‐bearing KPC mice were randomly divided into the treatment group and the control group. Prior to implantation, mice were anesthetized with 2% tribromoethanol (0.1 mL/body weight). A volume of 25 µL containing 2 MBq ^90^Y‐loaded or empty microspheres was injected into the visible lesion within the enlarged and deformed pancreas via laparotomy. PET/CT imaging was repeated 2 weeks after the procedure to assess SUVmax. All mice were provided food and water ad libitum. All mice were monitored according to IACUC guidelines and sacrificed if an excessive deterioration was observed. Tumor tissues were dissected and subjected to flow cytometry and IHC analyses.

### 5.9 Statistical Analysis

The diameters of lesions were calculated as the mean of two independent observers’ measurements. Statistical analysis was performed using R (version 4.2.3). Changes in blood parameters were reported as mean (range). Other measurements were expressed as mean ± standard error. A paired *t*‐test was used to compare the pre‐ and postoperative serum parameters. The differences among the three groups were analyzed using a one‐way ANOVA. Significance was assumed for *p* < 0.05.

## Author Contributions

Conception and design: B.C. and X.Z. Acquisition of the data: Y.Z., Y.Y., B.Z., Y.H., and D.Z. Drafting and revising of the manuscript: Y.Z., Y.Y., B.Z., H.C., and L.L. Review and editing: R.W., J.W., S.X., and S.B. Transporting of the radionuclide: W.M. and X.Z. The guarantors of the integrity of the study: B.C. and X.Z. All authors read and approved the final manuscript.

## Ethics Statement

This protocol and procedure in this study were approved by the ethics committee of Tongji Hospital of Tongji Medical College, Huazhong University of Science and Technology (TJH‐202206031). All animals were treated in accordance with the National Institutes of Health (USA) guidelines on the care and use of laboratory animals.

## Consent

The authors have nothing to report.

## Conflicts of Interest

W.L.M. and X.X.Z. are employees of Chengdu New Radiomedicine Technology Company; the company had no role in this study. All other authors declare no conflicts of interest.

## Supporting information



Supporting Information

Supporting Information

## Data Availability

The data used and/or analyzed during the current study is available from the corresponding author on reasonable request.

## References

[1] T. F. Stoop , R. T. Theijse , L. W. F. Seelen , et al., “Preoperative Chemotherapy, Radiotherapy and Surgical Decision‐Making in Patients With Borderline Resectable and Locally Advanced Pancreatic Cancer,” Nature Reviews Gastroenterology & Hepatology 21, no. 2 (2024): 101–124. 38036745 10.1038/s41575-023-00856-2

[2] E. Versteijne , M. Suker , K. Groothuis , et al., “Preoperative Chemoradiotherapy Versus Immediate Surgery for Resectable and Borderline Resectable Pancreatic Cancer: Results of the Dutch Randomized Phase III PREOPANC Trial,” Journal of Clinical Oncology 38, no. 16 (2020): 1763–1773. 32105518 10.1200/JCO.19.02274 PMC8265386

[3] C. N. Hurt , S. Falk , T. Crosby , et al., “Long‐Term Results and Recurrence Patterns From SCALOP: A Phase II Randomised Trial of Gemcitabine‐ or Capecitabine‐Based Chemoradiation for Locally Advanced Pancreatic Cancer,” British Journal of Cancer 116, no. 10 (2017): 1264–1270. 28376080 10.1038/bjc.2017.95 PMC5482737

[4] S. Krishnan , A. S. Chadha , Y. Suh , et al., “Focal Radiation Therapy Dose Escalation Improves Overall Survival in Locally Advanced Pancreatic Cancer Patients Receiving Induction Chemotherapy and Consolidative Chemoradiation,” International Journal of Radiation and Oncology in Biology and Physics 94, no. 4 (2016): 755–765. 10.1016/j.ijrobp.2015.12.003 PMC4792191 26972648

[5] A. Torgeson , S. Lloyd , D. Boothe , et al., “Multiagent Induction Chemotherapy Followed by Chemoradiation Is Associated With Improved Survival in Locally Advanced Pancreatic Cancer,” Cancer 123, no. 19 (2017): 3816–3824. 28621885 10.1002/cncr.30780

[6] E. Versteijne , J. L. van Dam , M. Suker , et al., “Neoadjuvant Chemoradiotherapy Versus Upfront Surgery for Resectable and Borderline Resectable Pancreatic Cancer: Long‐Term Results of the Dutch Randomized PREOPANC Trial,” Journal of Clinical Oncology 40, no. 11 (2022): 1220–1230. 35084987 10.1200/JCO.21.02233

[7] C. M. Taniguchi , J. M. Frakes , T. A. Aguilera , et al., “Stereotactic Body Radiotherapy With or Without Selective Dismutase Mimetic in Pancreatic Adenocarcinoma: An Adaptive, Randomised, Double‐Blind, Placebo‐Controlled, Phase 1b/2 Trial,” The Lancet Oncology 24, no. 12 (2023): 1387–1398. 38039992 10.1016/S1470-2045(23)00478-3 PMC11955909

[8] M. de Scordilli , A. Michelotti , D. Zara , et al., “Preoperative Treatments in Borderline Resectable and Locally Advanced Pancreatic Cancer: Current Evidence and New Perspectives,” Critical Reviews in Oncology/Hematology 186 (2023): 104013. 37116817 10.1016/j.critrevonc.2023.104013

[9] M. H. G. Katz , Q. Shi , J. Meyers , et al., “Efficacy of Preoperative mFOLFIRINOX vs mFOLFIRINOX Plus Hypofractionated Radiotherapy for Borderline Resectable Adenocarcinoma of the Pancreas: The A021501 Phase 2 Randomized Clinical Trial,” JAMA Oncology 8, no. 9 (2022): 1263–1270. 35834226 10.1001/jamaoncol.2022.2319 PMC9284408

[10] Q. P. Janssen , J. L. van Dam , L. R. Prakash , et al., “Neoadjuvant Radiotherapy After (m)FOLFIRINOX for Borderline Resectable Pancreatic Adenocarcinoma: A TAPS Consortium Study,” Journal of the National Comprehensive Cancer Network: JNCCN 20, no. 7 (2022): 783–791.e781. 35830887 10.6004/jnccn.2022.7008 PMC9326480

[11] J. Naidu , D. Bartholomeusz , J. Zobel , et al., “Combined Chemotherapy and Endoscopic Ultrasound‐Guided Intratumoral 32P Implantation for Locally Advanced Pancreatic Adenocarcinoma: A Pilot Study,” Endoscopy 54, no. 1 (2022): 75–80. 33440437 10.1055/a-1353-0941

[12] R. L. J. van Wanrooij , M. Bronswijk , R. Kunda , et al., “Therapeutic Endoscopic Ultrasound: European Society of Gastrointestinal Endoscopy (ESGE) Technical Review,” Endoscopy 54, no. 3 (2022): 310–332. 35114696 10.1055/a-1738-6780

[13] M. Ashat , R. El‐Abiad , A. Shrigiriwar , and M. A. Khashab , “Interventional Endoscopic Ultrasound: Current Status and Future Frontiers,” American Journal of Gastroenterology 118, no. 10 (2023): 1768–1778. 37646335 10.14309/ajg.0000000000002487

[14] K. X. Wang , Z. D. Jin , Y. Q. Du , et al., “EUS‐Guided Celiac Ganglion Irradiation With Iodine‐125 Seeds for Pain Control in Pancreatic Carcinoma: A Prospective Pilot Study,” Gastrointestinal Endoscopy 76, no. 5 (2012): 945–952. 22841501 10.1016/j.gie.2012.05.032

[15] M. J. Levy , M. D. Topazian , M. J. Wiersema , et al., “Initial Evaluation of the Efficacy and Safety of Endoscopic Ultrasound‐Guided Direct Ganglia Neurolysis and Block,” American Journal of Gastroenterology 103, no. 1 (2008): 98–103. 17970834 10.1111/j.1572-0241.2007.01607.x

[16] P. J. Ross , H. S. Wasan , D. Croagh , et al., “Results of a Single‐Arm Pilot Study of (32)P Microparticles in Unresectable Locally Advanced Pancreatic Adenocarcinoma With Gemcitabine/Nab‐Paclitaxel or FOLFIRINOX Chemotherapy,” ESMO Open 7, no. 1 (2022): 100356. 34953400 10.1016/j.esmoop.2021.100356 PMC8717429

[17] Y. H. Gholami , N. Wilson , D. James , and Z. Kuncic , “Toward Personalized Dosimetry With (32)P Microparticle Therapy for Advanced Pancreatic Cancer,” International Journal of Radiation and Oncology in Biology and Physics 99, no. 4 (2017): 1029–1038. 10.1016/j.ijrobp.2017.07.031 29063838

[18] G. N. Cohen , J. J. Munro 3rd , A. Kirov , et al., “32P Brachytherapy Conformal Source Model RIC‐100 for High‐Dose‐Rate Treatment of Superficial Disease: Monte Carlo Calculations, Diode Measurements, and Clinical Implementation,” International Journal of Radiation and Oncology in Biology and Physics 88, no. 3 (2014): 746–752. 10.1016/j.ijrobp.2013.11.006 24411623

[19] R. Salem , A. C. Gordon , S. Mouli , et al., “Y90 Radioembolization Significantly Prolongs Time to Progression Compared With Chemoembolization in Patients With Hepatocellular Carcinoma,” Gastroenterology 151, no. 6 (2016): 1155–1163.e1152. 27575820 10.1053/j.gastro.2016.08.029 PMC5124387

[20] V. Vilgrain , H. Pereira , E. Assenat , et al., “Efficacy and Safety of Selective Internal Radiotherapy With Yttrium‐90 Resin Microspheres Compared With Sorafenib in Locally Advanced and Inoperable Hepatocellular Carcinoma (SARAH): An Open‐Label Randomised Controlled Phase 3 Trial,” Lancet Oncology 18, no. 12 (2017): 1624–1636. 29107679 10.1016/S1470-2045(17)30683-6

[21] J. L. Schaal , J. Bhattacharyya , J. Brownstein , et al., “Brachytherapy via a Depot of Biopolymer‐Bound (131)I Synergizes With Nanoparticle Paclitaxel in Therapy‐Resistant Pancreatic Tumours,” Nature Biomedical Engineering 6, no. 10 (2022): 1148–1166. 10.1038/s41551-022-00949-4 PMC10389695 36261625

[22] J. Bai , Q. Yu , Y. Wang , et al., “Iodine‐125 Brachytherapy Suppresses Tumor Growth and Alters Bone Metabolism in a H1299 Xenograft Mouse Model,” Medical Oncology (Northwood, London, England) 40, no. 2 (2023): 72. 36607460 10.1007/s12032-022-01937-z

[23] M. Palta , D. Godfrey , K. A. Goodman , et al., “Radiation Therapy for Pancreatic Cancer: Executive Summary of an ASTRO Clinical Practice Guideline,” Practical Radiation Oncology 9, no. 5 (2019): 322–332. 31474330 10.1016/j.prro.2019.06.016

[24] W. A. Hall , L. A. Dawson , T. S. Hong , et al., “Value of Neoadjuvant Radiation Therapy in the Management of Pancreatic Adenocarcinoma,” Journal of Clinical Oncology 39, no. 34 (2021): 3773–3777. 34623894 10.1200/JCO.21.01220 PMC8608256

[25] P. Ghaneh , D. Palmer , S. Cicconi , et al., “Immediate Surgery Compared With Short‐Course Neoadjuvant Gemcitabine Plus Capecitabine, FOLFIRINOX, or Chemoradiotherapy in Patients With Borderline Resectable Pancreatic Cancer (ESPAC5): A Four‐Arm, Multicentre, Randomised, Phase 2 Trial,” Lancet Gastroenterology & Hepatology 8, no. 2 (2023): 157–168. 36521500 10.1016/S2468-1253(22)00348-X

[26] J. Ye , N. W. Gavras , D. C. Keeley , et al., “CD73 and PD‐L1 Dual Blockade Amplifies Antitumor Efficacy of SBRT in Murine PDAC Models,” Journal for Immunotherapy of Cancer 11, no. 5 (2023): e006842. 37142292 10.1136/jitc-2023-006842 PMC10163599

[27] C. W. F. van Eijck , D. A. M. Mustafa , D. Vadgama , et al., “Enhanced Antitumour Immunity Following Neoadjuvant Chemoradiotherapy Mediates a Favourable Prognosis in Women With Resected Pancreatic Cancer,” Gut 73, no. 2 (2024): 311–324. 37709493 10.1136/gutjnl-2023-330480 PMC10850691

[28] V. Chew , Y. H. Lee , L. Pan , et al., “Immune Activation Underlies a Sustained Clinical Response to Yttrium‐90 Radioembolisation in Hepatocellular Carcinoma,” Gut 68, no. 2 (2019): 335–346. 29440463 10.1136/gutjnl-2017-315485 PMC6352403

[29] S. P. Kim , C. Cohalan , N. Kopek , and S. A. Enger , “A Guide to (90)Y Radioembolization and Its Dosimetry,” Physical Medicine 68 (2019): 132–145. 10.1016/j.ejmp.2019.09.236 31785502

[30] W. Chen , W. Peng , R. Wang , et al., “Exosome‐Derived tRNA Fragments tRF‐GluCTC‐0005 Promotes Pancreatic Cancer Liver Metastasis by Activating Hepatic Stellate Cells,” Cell Death & Disease 15, no. 1 (2024): 102. 38291031 10.1038/s41419-024-06482-3 PMC10827722

[31] T. N. Fujimoto , L. E. Colbert , Y. Huang , et al., “Selective EGLN Inhibition Enables Ablative Radiotherapy and Improves Survival in Unresectable Pancreatic Cancer,” Cancer Research 79, no. 9 (2019): 2327–2338. 31043430 10.1158/0008-5472.CAN-18-1785 PMC6666414

