# Supplementary material for: Endoscopic Ultrasound‐Guided Brachytherapy of Yttrium‐90 Implantation Into Pancreas: A Dose‐Escalation Pilot Study
Source: MedComm (2020). 2025 Feb 24;6(3):e70117. doi: 10.1002/mco2.70117 (PMC11850441; doi:10.1002/mco2.70117)
Supplement: Supplementary file 1 — Supporting Information [file MCO2-6-e70117-s002.pdf]

Supplementary Materials

Endoscopic ultrasound-guided brachytherapy of Yttrium-90 implantation into pancreas: a dose-escalation pilot study

Running title: EUS-guided brachytherapy of ^{90}Y implantation

Yuchong Zhao MD [1, #], Yilei Yang MD [1, #], Buchuan Zhang PhD [2, #], Haochen Cui MD [1], Luyao Liu MS [1], Ronghua Wang PhD [3], Yunfeng Han, MD [2], Dongling Zhu, MD [2], Wenliang Ma MS [4], Xinxing Zhang MS [4], Jinlin Wang MD [5], Si Xiong PhD [1], Shuya Bai PhD [1], Xiaohua Zhu PhD [2, *], Bin Cheng MD [1, *]

[1] Department of Gastroenterology and Hepatology, Tongji Hospital, Tongji Medical College, Huazhong University of Science and Technology, Wuhan 430030, China.
[2] Department of Nuclear Medicine, Tongji Hospital, Tongji Medical College, Huazhong University of Science and Technology, Wuhan 430030, China.
[3] Department of Surgery, University of Pittsburgh School of Medicine, Pittsburgh PA 15213M, USA.
[4] Chengdu New Radiomedicine Technology Co., Ltd, Chengdu 610200, China.
[5] Department of Oncology, Tongji Hospital, Tongji Medical College, Huazhong University of Science and Technology, Wuhan 430030, China.

[#] Drs. Zhao, B. Zhang, and Yang contributed equally to this work.
[*] Corresponding to Bin Cheng, b.cheng@tjh.tjmu.edu.cn; Xiaohua Zhu, evazhu@vip.sina.com

Acknowledgments
This work was supported by the National Natural Science Foundation of China (No.82203812, No.82173318).

Supplementary Figures

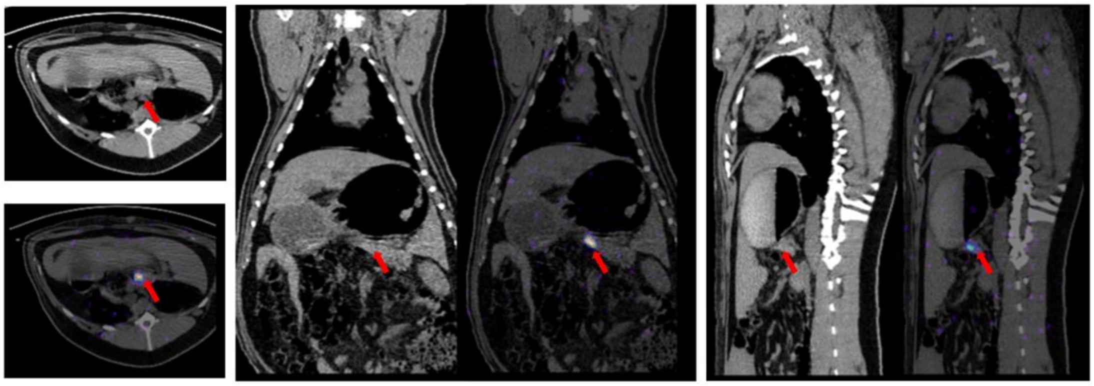

Figure S1: Postoperative PET/CT imaging of pig No.9 (60 MBq). The injection site in pancreas showed high uptake 3 days postoperatively and no uptake signal was observed in other areas. (pancreas: red arrow) (left: transverse plane; middle: coronal plane; right: sagittal plane).

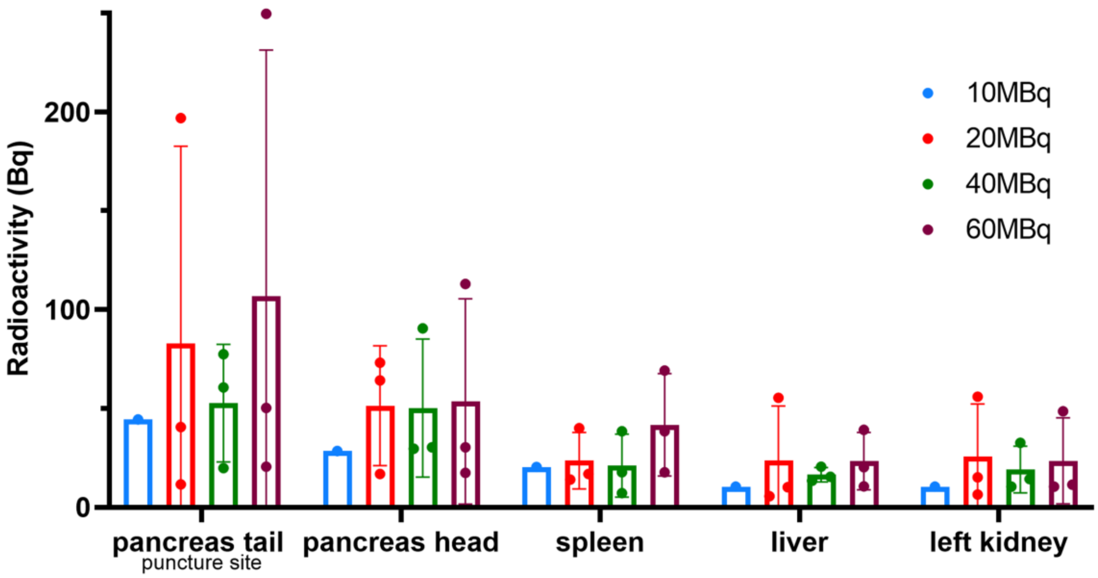

Figure S2: Radioactivity monitoring of adjacent organs. The radioactivity was slightly elevated in pancreas tail and the radioactivity in pancreas head and surrounding organs was not higher than the environmental dose.

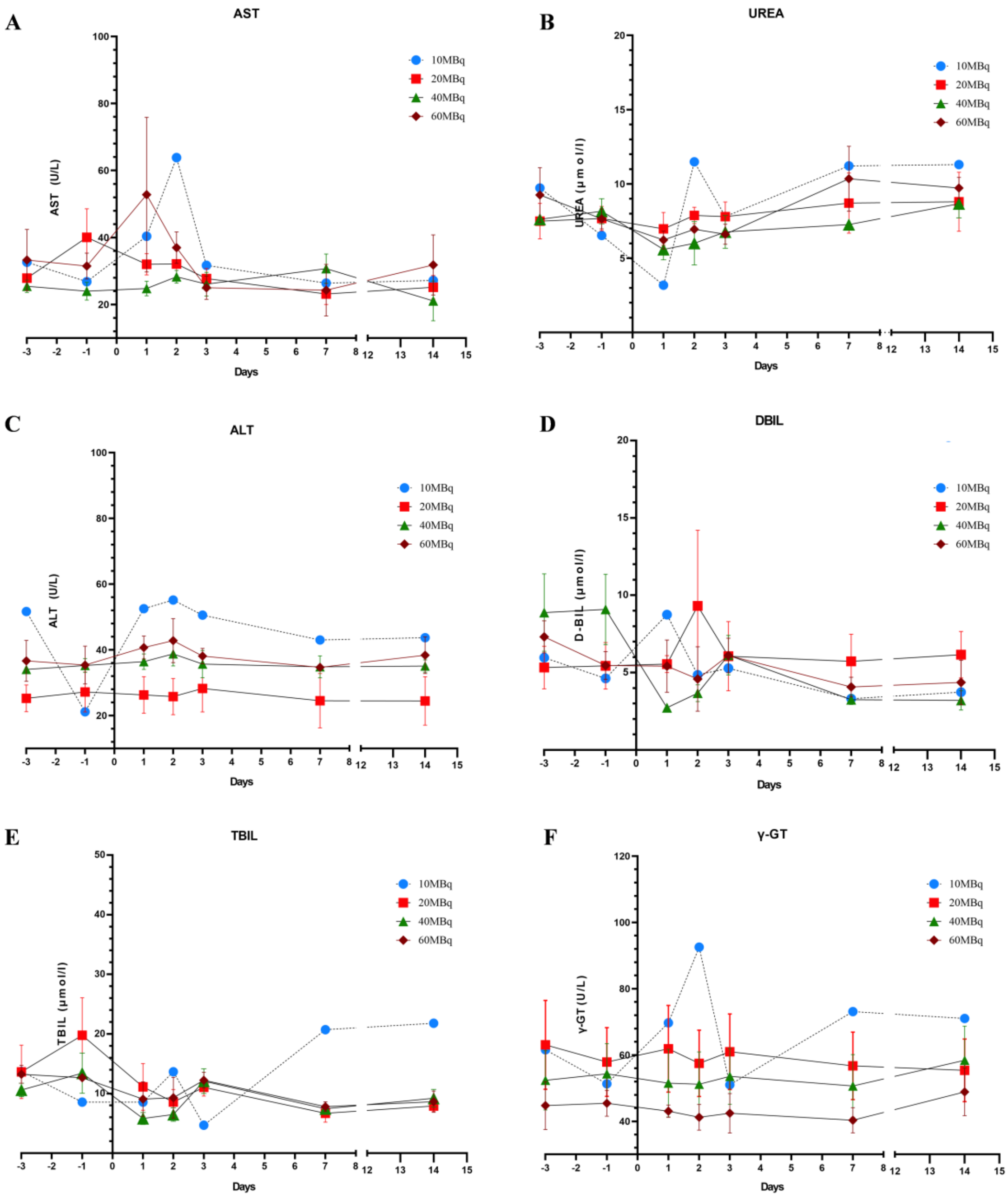

Figure S3: Serum index monitoring. Moderated elevation of AST to 98.30 U/L (3.8 times to the preoperative baseline) on Day 1 postoperative was observed in one pig (**a**). The postoperative ALT, TBIL, DBIL, γ-GT, GLU, and UREA were in the normal range (**b** – **e**).

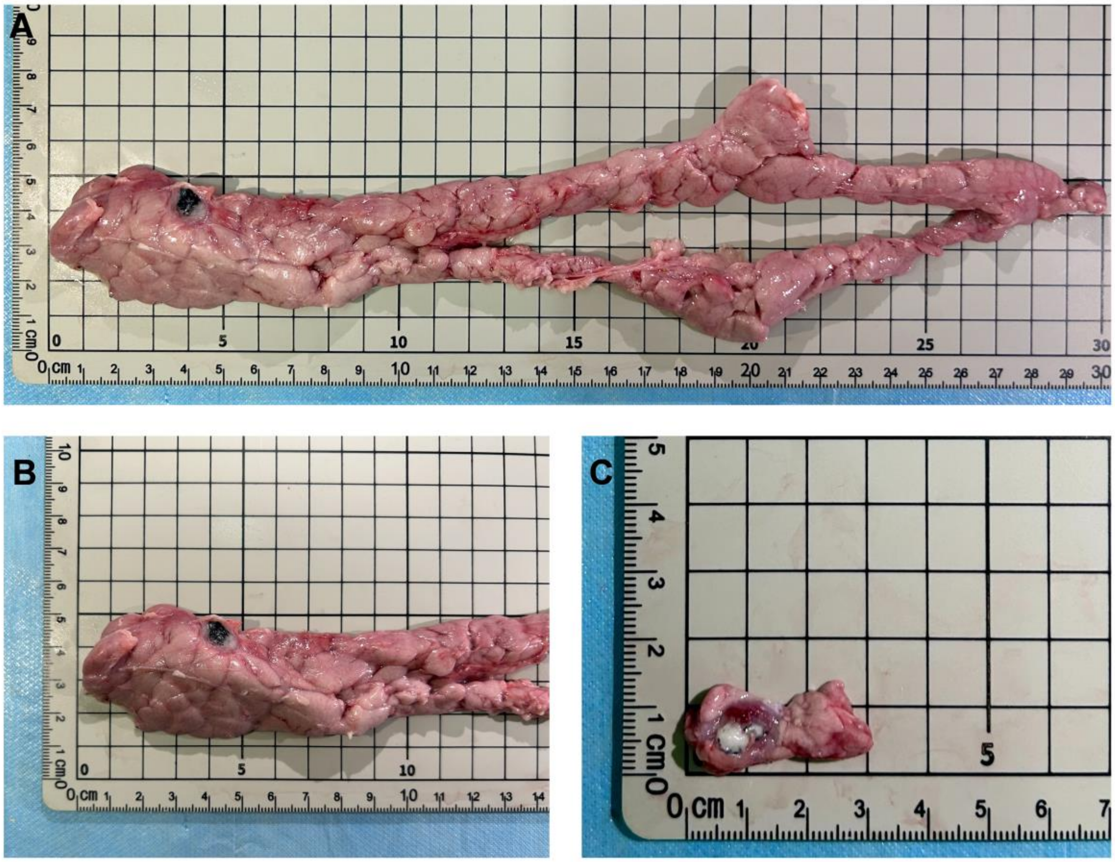

Figure S4: Excised pancreas. a – b. A visible necrotic area on the pancreas surface. **c.** A gray-white area with a black peripheral rim distinguishes necrotic area from healthy pancreas tissue.

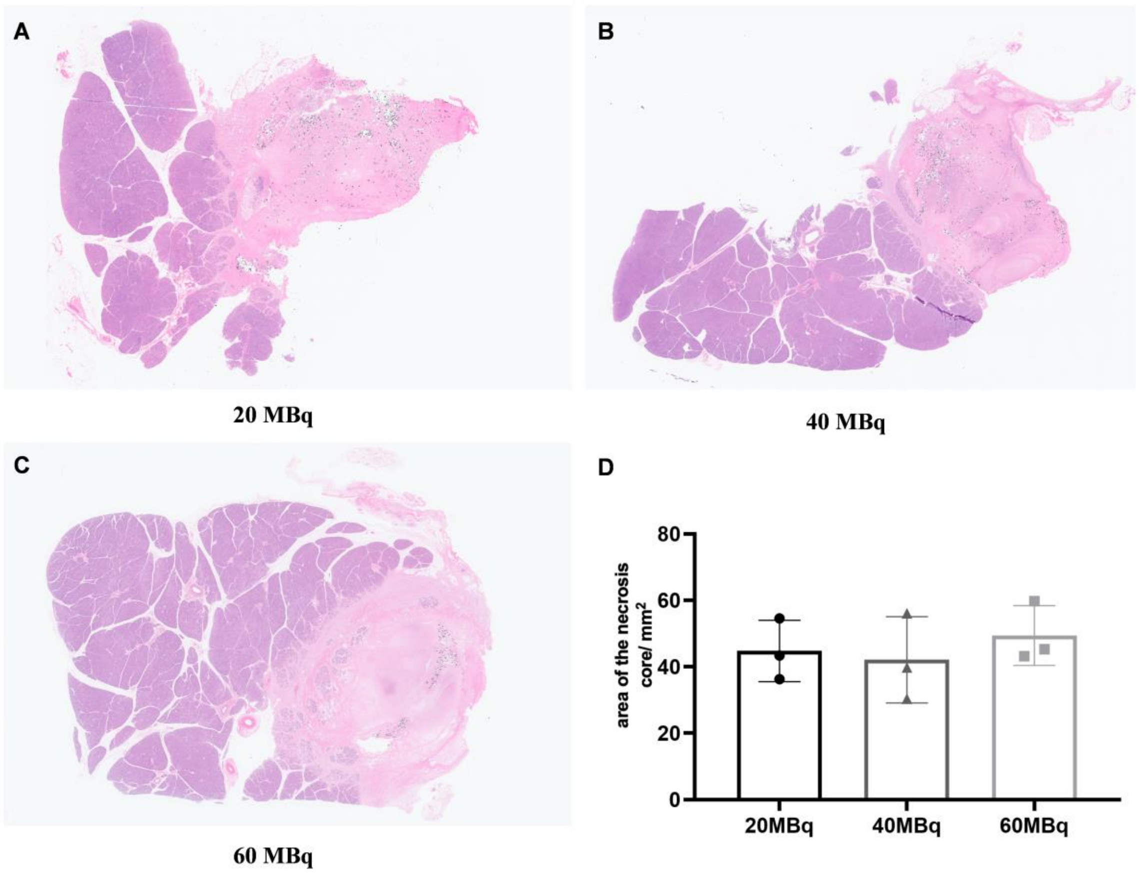

Figure S5: Radiation-induced necrotic area in pigs of each group. Representative necrosis in 20 MBq (**a.** pig No.4), 40 MBq (**b.** pig No.6), and 60 MBq (**c.** pig No.10) group. There were no significant differences of necrotic area among each group.

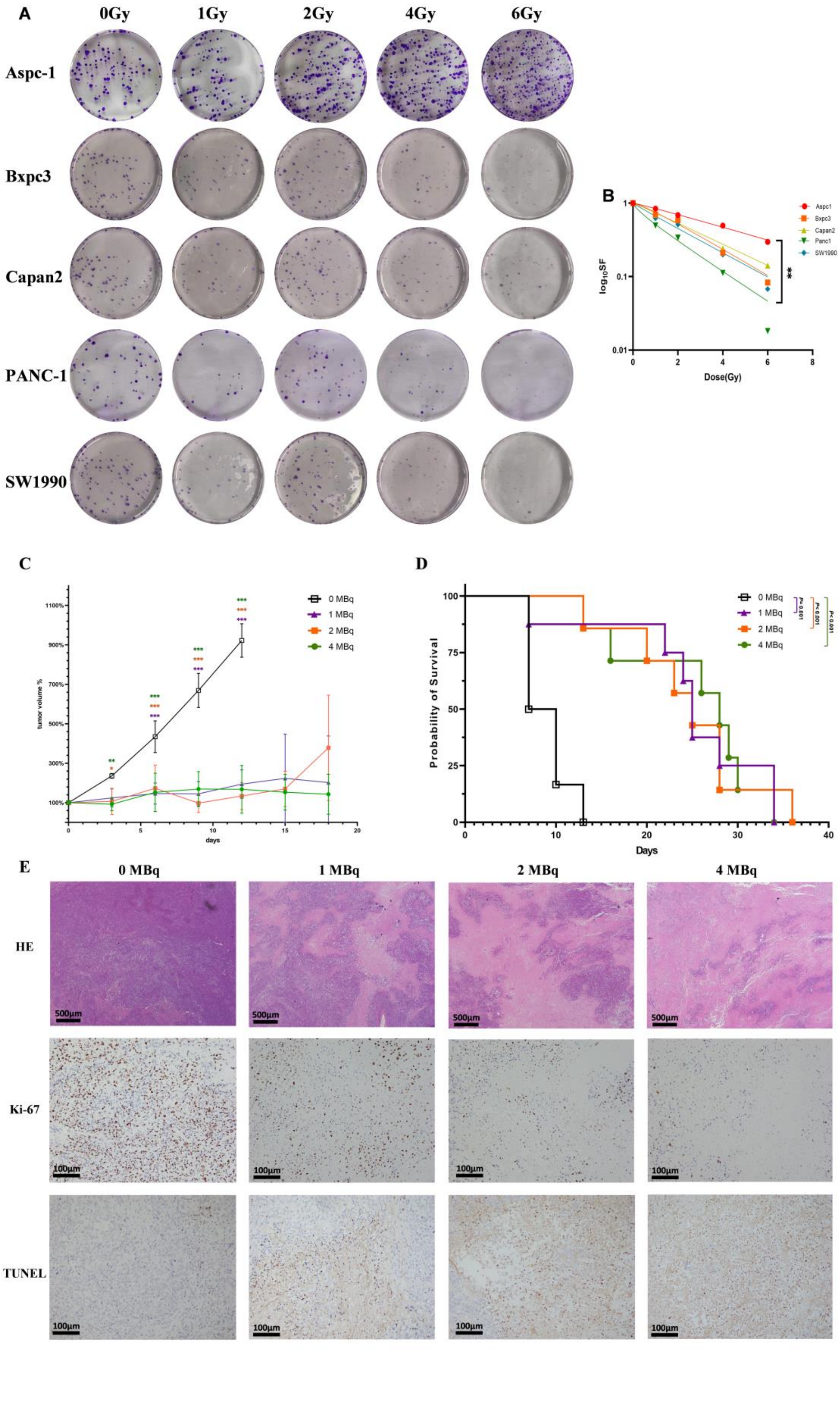

A
0Gy
1Gy
2Gy
4Gy
6Gy
Aspc-1
Bxpc3
Capan2
PANC-1
SW1990
B
log10SF
Dose(Gy)
Aspc1
Bxpc3
Capan2
Panc1
SW1990
**
C
tumor volume %
days
0 MBq
1 MBq
2 MBq
4 MBq
D
Probability of Survival
Days
0 MBq
1 MBq
2 MBq
4 MBq
P< 0.001
P< 0.001
P< 0.001
E
0 MBq
1 MBq
2 MBq
4 MBq
HE
Ki-67
TUNEL
500μm
100μm

Figure S6: Assessment of the effect of ^{90}Y in AsPC-1 xenograft model. a. radiation clonogenic assay of 5 types of pancreatic cancer cell lines. **b.** survival fraction with multi-target single-hit model. **c.** ^{90}Y implantation suppressed the growth of pancreatic cancer cell line AsPC-1 xenografts in nude mice. **d.** ^{90}Y implantation significantly prolonged the OS of nude mice in three treatment groups compared with the control. **e.** No complete responses were observed in the AsPC-1 xenograft model. High dose of radiation was correlated with a decreased ki-67 index and increased apoptosis. $^{**}P < 0.01$, $^{***}P < 0.001$.

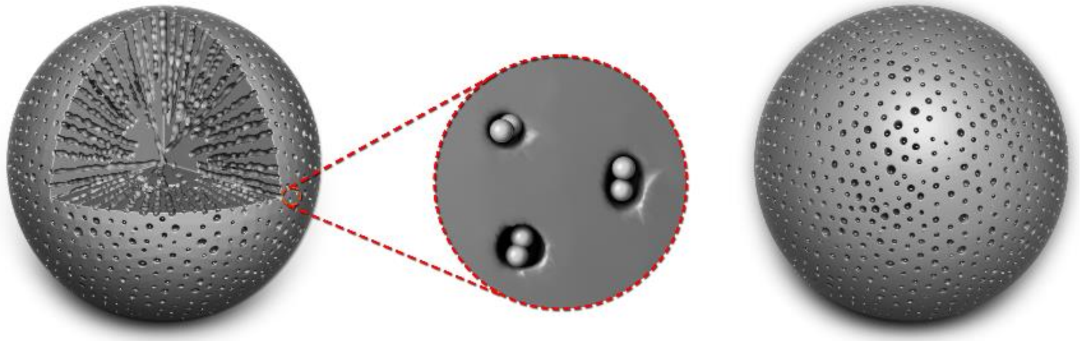

Figure S7: A model of the ^{90}Y-loaded carbon microspheres. Yttrium-90 phosphate was carried by a 32 μm (mean diameter) porous microsphere.

Supplementary Tables

Table S1: Detection of leakage into blood, urine, and stools

Activity	Pre-Day 1	Post 1 hour	Post 3 hours	Post-Day 1	Post-Day 2	Post-Day 3	Post-Day 7	Post-Day 14
Blood, Bq (SD)	0.65, 0.10	0.12, 0.15	0.08, 0.05	0.60, 0.42	2.20, 0.42	0.20, 0.11	0.05, 0.06	0.00. 0.00
Urine, Bq (SD)	0.87, 0.12	-	-	6.72, 8.46	7.77, 8.91	1.08, 0.75	1.95, 1.70	0.23, 0.33
Stool, Bq (SD)	1.92, 1.04	-	-	0.08, 0.10	7.96, 15.74	0.43, 0.16	1.05, 1.11	0.13, 0.05

SD, standard deviation

Table S2. The radiosensitivity of pancreatic cancer cell lines calculated survival fraction using the multi-target single-hit model

Cell line	D0	N
AsPC-1	4.537 ± 0.243**	1.127 ± 0.038
BxPC3	2.337 ± 0.030	1.398 ± 0.066
SW1990	2.619 ± 0.0850	0.959 ± 0.007
Capan2	2.993 ± 0.055**	1.071 ± 0.004
PANC-1	2.303 ± 0.126	0.710 ± 0.013

**P<0.01 *vs.* PANC-1

Table S3. Injection dose of each pig

	No. of pig	Calibration activity (MBq)	Duration from calibration to injection (min)	Residual activity (MBq)	Estimated injection activity (MBq)
Initial dose	No. 1	14.7	3.3	4.0	10.7
	No. 2	23.2	8.4	3.7	19.5
Low dose	No. 3	28.0	8.9	8.2	19.1
	No. 4	26.0	8.2	7.4	19.6
	No. 5	48.0	9.6	7.1	40.9
Medium dose	No. 6	49.0	7.2	8.7	40.3
	No. 7	54.0	1.2	8.8	45.2
	No. 8	69.0	3.3	4.5	64.5
High dose	No. 9	63.0	6.0	5.0	58.0
	No. 10	67.0	6.6	8.0	59.0
